# Prostaglandins in the pathogenesis of kidney diseases

**DOI:** 10.18632/oncotarget.25005

**Published:** 2018-05-29

**Authors:** Yuanyuan Li, Weiwei Xia, Fei Zhao, Zhaoying Wen, Aihua Zhang, Songming Huang, Zhanjun Jia, Yue Zhang

**Affiliations:** ^1^ Department of Nephrology, Children’s Hospital of Nanjing Medical University, Nanjing 210008, China; ^2^ Jiangsu Key Laboratory of Pediatrics, Nanjing Medical University, Nanjing 210029, China; ^3^ Nanjing Key Laboratory of Pediatrics, Children’s Hospital of Nanjing Medical University, Nanjing 210008, China

**Keywords:** prostaglandins, kidney, acute kidney injury, chronic kidney injury

## Abstract

Prostaglandins (PGs) are important lipid mediators produced from arachidonic acid via the sequential catalyzation of cyclooxygenases (COXs) and specific prostaglandin synthases. There are five subtypes of PGs, namely PGE2, PGI2, PGD2, PGF2α, and thromboxane A2 (TXA2). PGs exert distinct roles by combining to a diverse family of membrane-spanning G protein-coupled prostanoid receptors. The distribution of these PGs, their specific synthases and receptors vary a lot in the kidney. This review summarized the recent findings of PGs together with the COXs and their specific synthases and receptors in regulating renal function and highlighted the insights into their roles in the pathogenesis of various kidney diseases.

## INTRODUCTION

Diminished kidney function is commonly classified as two distinct syndromes named chronic kidney disease (CKD) and acute kidney injury (AKI) [1]. CKD is a global public health issue, and its prevalence is estimated to be 8–16% worldwide [2]. The prevalence and mortality of AKI is also increasing all over the world, especially in developing countries. Due to the different definitions of AKI, wide variation exists in the estimation of disease prevalence (1–25%) and mortality (15–60%) [3, 4]. Although massive researches have been performed in the past decades, the pathogenic mechanisms of CKD and AKI are still elusive. Moreover, to date, no specific therapies have emerged to lessen the incidence or assist the recovery of kidney diseases. Effective treatment approaches are urgently needed to be developed.

**Figure 1:**
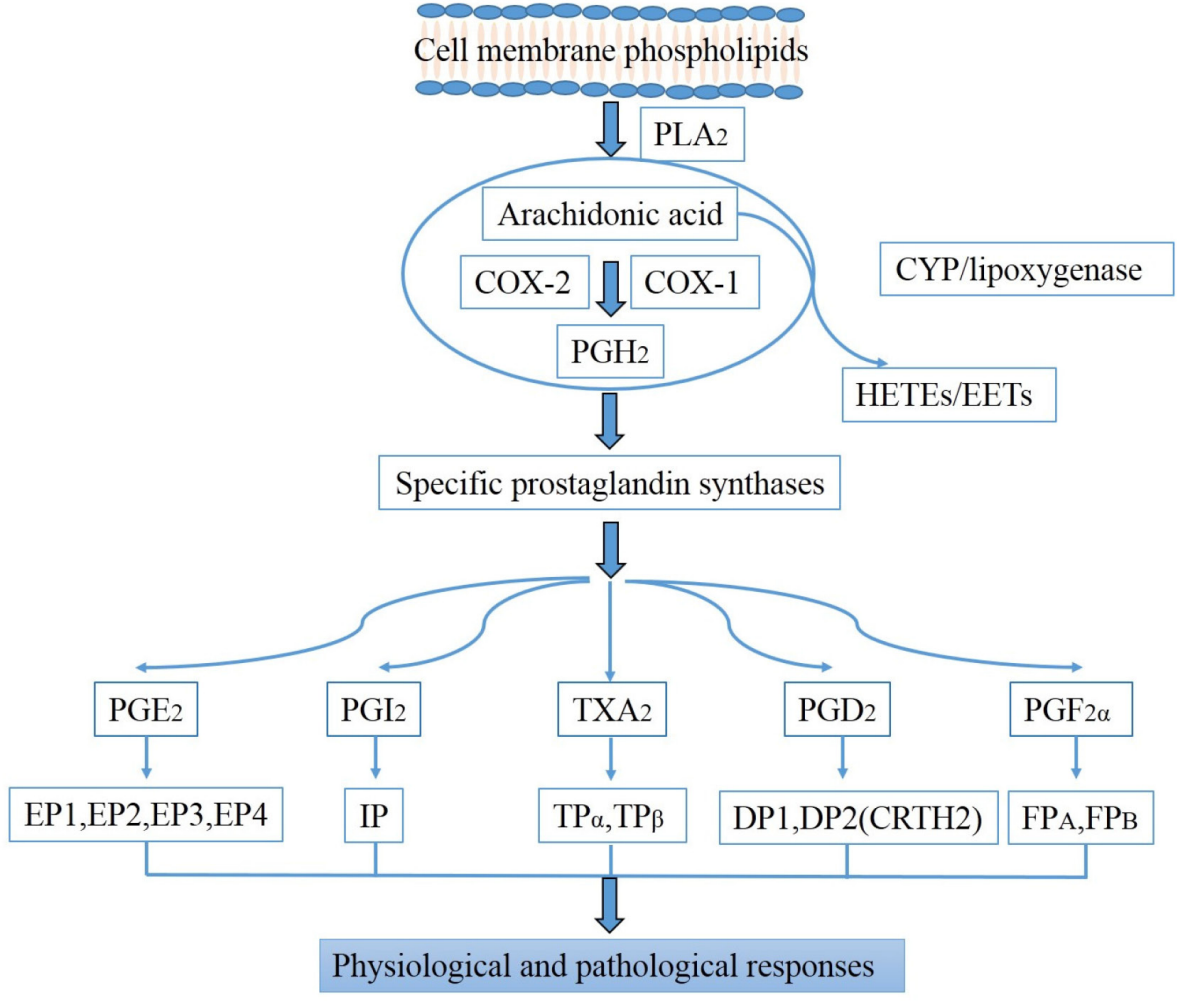
Biosynthesis pathways of prostaglandins

Inflammation is the established causative factor in the kidney diseases [5]. PGs are abundantly produced in the kidney (Table 1), and play an important role in triggering the inflammatory response, contributing to the occurrence and progression of kidney diseases [6–8]. PGs appear when arachidonic acid (AA) is released from the plasma membrane by phospholipases and metabolized by the peroxidase actions of COXs to PGH_2_ which can be subsequently converted into more stable biologically active PGs, including PGE2, PGI2, PGD2, PGF2α, and TXA2 by their respective synthases [7, 9] (Figure 1). PGs exert their functions by combining to a diverse family of membrane-spanning G protein-coupled prostanoid receptors [10, 11] (Table 2).

**Table 1:** Renal distributions of catalytic enzymes of PGs

Enzymes		Distribution
COX-1		Collecting duct, glomeruli, medullary interstitial cells, arterial endothelial cells
COX-2		Medullary interstitial cells, glomeruli, macula densa, thick ascending limb,
PGES	mPGES-1	Macula densa, distal convoluted tubule, collecting duct, and renal medullary interstitial cells
	mPGES-2	Distal convoluted tubule, collecting duct, proximal convoluted tubule, thick limbs of the loops of Henle
	cPGES	All nephron segments
PGIS		Glomeruli, medullary collecting duct.
TXAS		Glomeruli
PGDS		Proximal convoluted tubule, thick ascending limb, distal convoluted tubule, collecting duct
PGF2α synthase		Not defined

**Table 2:** Renal functions, possible signaling pathways, pharmacological modulators, and renal distributions of PG receptors

PG receptors		Renal function	Signaling pathways	Agonist/Antagonist	Expressionsites
EP	EP1	Haemodynamics, transport, proliferation, fibrosis, Renin release	Gq-Ca^2+^	A: Sulprostone;17-phenyl trinor prostaglandin E_2_ethyl amid	CD/MG/P/PT/V
				Ant: ONO-8711, ONO-8713	
	EP2	Haemodynamics, transport, anti-apoptosis, Renin release	Gs-cAMP, adenylatecyclase, β-arrestin mediated signalosome	A: CP-536, 745-01; butaprost	IC/MD/P/V
	EP3	Transport, vasoconstriction	Gi-cAMP, adenylate cyclase, G12/13-RhoA	A: L-798106; sulprostone	DT/CD/MD/V
	EP4	Haemodynamics, transport, renin release, vasodilation, proliferation, anti-apoptosis	Gs-cAMP, Gi-cAMP, adenylate cyclase, β-arrestin mediated signalosome	A: CP-044, 519-02; 11-deoxy-PGE1; cay10580,	P/CD/MG/MD/DT/PT/V
				Ant: ONO-AE3-208; L-161982	
IP		Haemodynamics, vasodilation, transport, renin release, matrix synthesis, proliferation, anti-apoptosis	Gs-cAMP	A: ONO-1301; MRE-269	MG/MD/DT/CD/PT/P/V
TP		Vasoconstriction, haemodynamics, fibrosis, proliferation, differentiation, inflammation	Gq-Ca2+, G12/13-RhoA	A: U-46619	MG/P/V/DT/CD/PT
				Ant: S18886	
DP		Fibrosis, inflammation, haemodynamics, transport	Gs-cAMP	Ant: CAY10471	--
FP		Transport and cell transformation, haemodynamics, growth	Gq-Ca2+, Gi-cAMP, G12/13-RhoA	A: Latanoprost	CD/DT/P/F

Abbreviations: CD: collecting duct; MG: mesangial cells; P: podocytes; PT: proximal tubule; DT: distal tubule; V: vasculature; F: fibroblasts; IC: interstitial cells; MD: macula densa; A: agonist; Ant: antagonist.

PGs are important lipid mediators in numerous physiological and pathophysiological processes in the kidney. Under the physiological conditions, PGs exert essential functions in the regulation of renal hemodynamics, renin release, as well as water and salt balance [7, 12, 13]. Likewise, PGs also respond to distinct pathological insults. Researches on the functions of PGs in CKD and AKI have been conducted and the awareness have been drawn. In this review, we highlighted the roles of PGs, PG synthases, and the specific PG receptors in kidney diseases, with expectation to develop effective therapies targeting the PG cascades to relieve kidney injury.

## COX_S_ IN KIDNEY DISEASES

COXs are the rate-limiting enzymes that are responsible for the first two steps in the synthesis of PGs. The production of PGs is depended on the activity of COXs which are the upstream components of this process. The COXs have both COX and peroxidase activities and exist as two isoforms termed as COX-1 and COX-2 [9, 14]. COX-1 is constitutive as its abundant expression in a wide range of tissues under basal conditions. In the kidney, COX-1 highly expresses in the collecting duct, while lower level of COX-1 can be detected in interstitial cells, glomerular mesangial cells, and endothelial cells of arterioles [12, 15–17]. It is commonly recognized that COX-1 serves a constitutive housekeeping role [18]. Evidence has shown that COX-1 could regulate nephron formation via PGE2 in the zebrafish embryonic kidney [19]. COX-1 is also responsible for maintaining basic physiological functions including cytoprotection of the gastric mucosa and platelet aggregation [20, 21]. Furthermore, COX-1 functions as the major form rather than COX-2 in endothelium-dependent contraction [22, 23] or relaxation [24]. Beyond the scope of above discoveries, there are also many investigations focusing on kidney diseases [25–27]. Different from COX-1, COX-2 can be induced by inflammatory mediators and mitogens. It mediates the induction of various PGs, which play critical roles in various pathophysiological processes such as angiogenesis, inflammation and tumorigenesis [20, 28]. Whereas, several studies suggested that COX-2 also has housekeeping functions in kidney development [29, 30], ovulation, and parturition [31, 32]. In the kidney, COX-2 predominantly expresses in the macula densa, thick ascending limb, medullary interstitial cells, and glomerulus, contributing to a variety of physiological processes [12, 33]. Because of the specific distribution in the kidney, COX-2 is of vital importance in regulating renin release and water/salt metabolism [34]. More importantly, the significant role of COX-2 in kidney diseases, especially in CKD, has been documented by using COX-deficient mouse models or COX antagonists [35–42]. It is known that inhibition of COX-2 is associated with AKI incidence due to the imbalance of renal function in fluid excretion and intra-renal hemodynamics. Some investigations pointed out that suppression of COX-2 activity in AKI accounts for a depravation of renal function [43]. On the contrary, there were also some diverse results with the treatment of COX-2 inhibitor [8, 44, 45]. These diverse outcomes of COX-2 inhibition in AKI may be resulted from distinct experimental conditions such as various kinds of inhibitors and the distinction of disease models and animal species/strains. In addition, recent studies revealed that COX-2/PGE2 cascade activation mediated cisplatin-induced mesangial cell apoptosis and chronic renal failure of C57/BL6 mice with 5/6 nephrectomy [46, 47]. Apart from COXs, arachidonic acid can be metabolized via lipoxygenase and cytochrome P-450 (CYP) enzymes to some other metabolites. Early researches demonstrated the role of 20-HETE, one metabolite of arachidonic acid converted by cytochrome P-450 (CYP) enzyme, in mediating the effects on tubuloglomerular feedback [48]. 20-HETE is also consistent with the known effects of NO, both of which are the main regulators of tubuloglomerular feedback [49]. NO inhibits the production of 20-HETE, which contributes to the vasodilator effect of NO [50–52]. Strategy targeting COXs could be effective because many evidence support a significant role of COXs in the progress of kidney diseases. However, severe side effects possibly due to the nonselective antagonism on the downstream metabolites of COXs greatly limited its application. Thus, selective inhibition of the specific PG synthetic enzymes might be more promising in treating kidney diseases.

## PGE2 IN KIDNEY DISEASES

It has been universally recognized that PGE2 is the most abundant renal arachidonic acid metabolite. PGE2 is produced by PGE synthases, and functions by combining to its G protein-coupled receptors: namely EP1, EP2, EP3, and EP4. Three PGE2 synthases in the kidney have been uncovered: microsomal PGE synthase 1 (mPGES-1), microsomal PGE synthase 2 (mPGES-2) and cytosolic PGE synthase (cPGES). Among these three PGESs, mPGES-1 is the best-characterized PGES. Its expression is inducible in the kidney in response to various stresses [53]. mPGES-1 expression can be detected in macula densa, distal convoluted tubule, collecting duct, and renal medullary interstitial cells. Studies revealed that mPGES-1 co-expresses with COX-1 and COX-2 in the kidney [7, 9]. Meanwhile, it has been indicated that mPGES-1 has the best PGE2 synthetic property both *in vivo* and *vitro* [5, 54, 55]. Additionally, recent studies have also shown that deletion of mPGES-2 or cPGES in mice did not reduce PGE2 levels [56]. The aggravation of AKI by downregulating mPGES-2was demonstrated to be associated with autophagy inhibition and enhanced apoptosis [57]. Multiple researches uncovered a remarkable role of mPGES-1 deletion on PGE2 production in various models including endotoxemia- and cisplatin-induced kidney injury [58], lithium-induced NDI [59], Ang II- and DOCA-salt-induced hypertension [60], aldosterone escape [61], and unilateral ureteral obstruction [62]. Different from these observations, in the diabetic kidney disease model induced by STZ, three forms of PGES were unaltered in the kidney, and deletion of mPGES-1 did not suppress renal PGE2 production [63]. These diverse results on PGE2 induction suggest that beyond three known PGESs, additional PGE synthases might be existed to exert the function in producing PGE2 under some specific pathological conditions. Due to the critical role of PGE2 in mediating the inflammatory responses, mPGES-1 is considered to contribute to chronic and acute kidney injuries, which has been confirmed in many studies from our and other groups. For instance, in the CKD model of 5/6 nephrectomy, mPGES-1 invalidation alleviated the loss of renal function characterized by the attenuated accumulation of uremic toxins in circulation and improved creatinine clearance [64]. In obese db/db mice with type-2 diabetes, the level of mPGES-1 expression in glomeruli was remarkably higher, suggesting the potential contribution of mPGES-1 in glomerular diseases associated with type-2 diabetes [65]. In kidney cells, we also found that mPGES-1-derived PGE2 could activate Stat3 signaling to promote podocyte apoptosis [66], and the proliferation of mesangial cells triggered by uric acid and uremic toxin indoxyl sulfate was attenuated by silencing mPGES-1 [67, 68]. However, in a UUO model, mPGES-1 was shown to exert a protective effect against renal fibrosis and inflammation [62]. Moreover, in the type-1 diabetic model induced by STZ, both renal PGE2 production and glomerular injury were unaffected in mPGES-1 KO mice. All these studies suggested conflict roles of mPGES-1 in mediating the pathogenesis of CKD, which indicated that the role of mPGES-1 in CKD might depend on the insults. Similarly, diverse conclusions also exist in some AKI models. When mice were challenged with cisplatin, LPS and ischemia/reperfusion, respectively, mPGES-1 deletion only played a role in cisplatin experimental setting [44]. Although above researches indicated a complexity of mPGES-1 in various kidney events, as a specific downstream enzyme of PGE2 generation, mPGES-1 is suggested to be associated with fewer adverse effects than COX inhibitors. Thus, mPGES-1 is still a promising target for the treatment of kidney diseases [69]. However, more extensive investigations on the efficacy and safety of mPGES-1 inhibition in treating various kinds of kidney diseases are required.

The role of PGE2 in the kidney has been comprehensively studied. PGE2 functions via activating four subtypes of receptors (EP1, EP2, EP3, EP4). EP1 is mostly detected in the collecting duct, and exerts its main role in natriuresis and diuresis under physiological status [70–73]. Recently, one research suggested that PGE2 regulated arginine vasopressin synthesis by hypothalamic EP1 [74]. Meanwhile, EP1 receptor, instead of EP2, was reported to increase renin expression in M-1 CD cells [73, 75]. Apart from the collecting duct, EP1 can be also detected in glomerular mesangial cells [76, 77], podocytes [78], and proximal tubule cells [79, 80], suggesting the additional functions of EP1 besides the diuretic action. In mesangial cells, PGE2-induced hypertrophy and cell cycle arrest was reproduced by EP1 agonist sulprostone [76]. It was also proved that EP1 receptor could suppress the proliferation of mouse mesangial cells to alleviate the progression of proteinuria, glomerular hypertrophy, and mesangial expansion through a selective EP1 antagonist, ONO-8713 [77, 81]. In diabetic EP1 KO mice, diabetic hyperfiltration, albuminuria, and injury markers were all suppressed possibly through modulating renin-angiotensin system. *In vitro* experiments with ONO-8711, an EP1 antagonist, further suggested a fundamental role of EP1 in podocyte impairment [78]. In renal tubules, EP1 deletion reduced the expressions of fibrotic markers and preserved megalin expression in diabetic mice [79, 80]. However, the controversy still exists about the role of EP1 receptor in kidney diseases. There was evidence showing that nephritic mice lacking the EP1 had increased severity of renal impairment [82].

Although PGE2 receptors have been intensively studied in the kidney, not much is known about EP2 receptor. EP2 is mainly found in vascular and interstitial compartments of the kidney [83]. Researchers once used two models including mercury chloride model of acute renal failure and 5/6 nephrectomy model of chronic renal failure to identify the effect of EP2 [84]. By use of the EP2 agonist, CP-536,745-01, reduction of glomerular sclerosis, amelioration of tubulointerstitial injury and better tubular structure were observed in the nephrotoxic mercury chloride (HgCl (2)) rat model of acute kidney failure. [85]. In a different model, EP2 receptor mediated the cystogenesis by inhibiting the apoptosis of cystic epithelial cells [86]. Another study identified that EP2 and EP4 receptors contributed to the pro-inflammatory effects of PGE2 [87]. In podocytes, COX-2/ PGE2/EP2 axis has been revealed to play a pathogenic role in hyperfiltration-mediated albuminuria, as well as the progression of CKD [88]. Meanwhile, EP2 and EP4 receptors are important in promoting the progression of chronic kidney disease induced by TGF-β, while EP1 and EP3 play the opposite role [89].

Renal EP3 expresses in thick ascending limbs and collecting duct existing as different splice variants which determine the preference for G-protein coupling. EP3 splice variants inhibit adenylate cyclase via Gi protein and increase intracellular calcium and activate the G12/G13 pathway, which later leads to the activation of Rho kinase, accounting for the action of EP3 receptor in urinary concentration. EP3 receptor may exert its role by modulating vasopressin signaling in lithium-induced and post-obstructive polyuria [16]. Similarly, EP3 could also cause vasoconstriction in rat proximal interlobular arteries, which was proved by using Misoprostol, and was suggested to protect the kidney during diabetic hyperfiltration regardless of RAAS activation [90, 91]. Beneficial effects of EP3 were also identified by EP3 antagonist, L-798106, evidenced by the retardation of normalized blood urea nitrogen, normalized glomerular cell numbers, restored synaptopodin distribution and F-actin filament arrangement in glomeruli which is mediated by PGE2 [92]. However, in a diabetic mouse model induced by streptozotocin, EP3 was shown to inhibit the water reabsorption, and contributed to polyuria [93].

EP4 receptor is the best identified subtype of the EP receptors, and is abundant in almost all types of renal cells. In addition, EP4 signaling plays a variety of roles through cAMP effectors, and is able to activate the PI3k-dependent pathway, leading to the activation of MAPK signal pathway [94]. Emerging evidence showed that it could couple to G(i)α, phosphatidylinositol 3-kinase, β-arrestin, and β-catenin [95–97]. The renal distribution of EP4 receptor involves the glomeruli, renin-secreting juxta-glomerular granular cells, glomerular epithelial cells, distal convoluted tubules, and cortical collecting ducts [98]. Lots of evidence indicates the function of EP4 in podocytes [99–102]. EP4 deletion from podocytes ameliorated kidney injury in both 5/6 nephrectomy and diabetic CKD models [99, 100]. However, diverse outcomes were seen in another kidney injury model induced by Adriamycin, showing that EP4 deletion and EP4 antagonist (L-161982) did not attenuate podocyte injury [101]. In addition, a selective EP4 agonist (11-deoxy-PGE1) promoted glomerulosclerosis and tubulointerstitial fibrosis in STZ-induced diabetic mice, possibly through IL-6 [103]. Recent evidence also showed that EP4 inhibition (ONO-AE3-208) ameliorated proteinuria and glomerular scarring in rats subjected to renal mass resection [104]. In LPS-induced renal proximal tubule cell injury, EP4 inhibition also played critical roles in anti-inflammatory and anti-apoptosis processes [105]. However, there were still lots of conflicts over EP4 receptor in kidney diseases. Several researches showed the protective role of EP4 in decreasing epithelial cells apoptosis, preventing mesangial cell injury, and preventing tubulointerstitial fibrosis [87, 102, 106]. Apart from these, EP4 is also an important component in the maintenance of body water homeostasis. EP4 expressed in the distal convoluted tubule and the cortical collecting duct and could increase AQP2 membrane trafficking and phosphorylation to enhance water reabsorption possibly via both cAMP/PKA and extracellular signal-regulated kinase (ERK) pathways [107]. Furthermore, a recent research emphasized the capability of EP4 in urine concentrating and provided a new pathway of AVP/PGE2/EP4/PRR for the regulation of AQP2 expression [108]. Overall, PGE2 couples with four EP subtypes to exert multiple functions in the pathology and physiology of kidney. With the development of novel agonists/antagonists and the researches on their application in various kidney diseases, the clinical therapies on kidney diseases by targeting EPs are becoming more feasible.

## PGI2 IN KIDNEY DISEASES

Prostacyclin (PGI2) is one of the major products of COXs pathway, and is well known for its regulation on renal hemodynamics, tubular transport and renin release. Prostacyclin synthase (PGIS) is an atypical cytochrome p450 enzyme that generates prostacyclin (PGI) from prostaglandin H (PGH) derived from arachidonic acid by COXs [109]. It is a disappointment that the exact cellular localization of PGIS protein could not be identified yet, while the significant expression of PGIS mRNA was detected in the inner medullary tubules and medullary interstitial cells [110]. Similar to PGES, investigations of PGIS were carried out by use of kidney disease models and PGIS gene knockout mice. Previous reports showed that PGIS deficiency induced renal fibrosis along with the notable irregulation of renal hemodynamics, tubular atrophy, surface irregularities and cysts [111, 112]. Furthermore, overexpression of PGIS contributed to the renal protection against endotoxemia-related AKI [113]. In uremic mice, the reduction of PGI2 synthase activity could be prevented by MnTBAP, a synthetic ROS-scavenging enzyme superoxide dismutase (SOD). It was suggested that the defect of PGI2-generating pathway could be mediated by oxidative stress which was attributable to the progression of end-stage renal disease (ESRD) [114]. Besides kidney cases, PGI2 is also known for its beneficial effects during the stroke, thrombosis, atherosclerosis, and myocardial infarction [115, 116], which may indirectly affect the progression of kidney diseases. A previous review had shown the connections between PGI2 and renal growth, fibrotic response, and cell fate [112]. Recently, a lot of studies focused on Beraprost Sodium (BPS) to reveal the role of PGI2 in kidney diseases. As a prostacyclin analogue, BPS is a vasoactive substance that can expand renal vessels to increase renal blood flow, inhibit TXA2 synthesis, and prevent platelet aggregation and immune complex formation. It was also reported that BPS could prevent glomerular thrombosis to reduce proteinuria [117]. Similar results were also shown in some clinical researches [118–120]. In addition, in a diabetic kidney disease induced by STZ, BPS improved renal function possibly through the inhibition of oxidative stress and inflammation [121, 122].

Major renal functions of PGI2 are mediated by the cell-surface receptor termed IP. The localization of IP receptor in renal cells is considered to be controversial over species. IP was reported to be detected in mesangial cells, interstitial cells, the vasculature and the tubular epithelial cells (proximal tubule, mTAL and collecting duct) in rodent kidney [70, 123, 124]. In human kidney, the IP receptor was also detected in podocytes [125]. This potentially indicated differences in function of IP receptor in the specific parts of nephron [124]. IP receptor is found to play a significant role in maintaining renal hemodynamics, tubule transport, renin secretion, and reducing renal fibrosis and inflammation [125]. One recent research showed that prostaglandin I2 receptor agonism preserved beta-cell function and attenuated albuminuria through nephrin-dependent mechanisms [126]. ONO-1301, a novel nonprostanoid IP agonist, was used in models of type 1 diabetic nephropathy and UUO, and showed a therapeutic effect on treating diabetic nephropathy via inducing hepatocyte growth factor (HGF) to counteract TGF-β [127, 128]. ONO-1301 also ameliorated the renal lesions in type 2 diabetes by attenuating mesangial matrix accumulation, inflammation and oxidative stress through an IP receptor-mediated mechanism [129]. Interestingly, comparing with IP-knockout mice, PGIS knockout mice displayed additional glomerular, vascular and interstitial abnormalities, suggesting the contribution of other receptors in addition to IP [113]. Till now, lots of evidence indicated that PGI2 could activate peroxisome proliferator-activated receptor α (PPARα) or peroxisome proliferator-activated receptor δ (PPARδ) to protect tubular cells from apoptosis in AKI [130, 131]. In addition, recent studies lay special stress on a cyclic vasoactive peptide termed urotensin II which could induce the production of PGI2 in gentamicin-treated NRK-52E cells and protect renal cells through a PPARα-dependent mechanism [132]. Moreover, researchers figured out that L-carnitine could protect renal tubular epithelial cells in the experimental animal model induced by carboplatin by activating PGI2/ PPARα signal pathway [133]. Taken together, PGI2, as an important product of COXs, plays an important role by coupling with its receptors and the downstream signals in various types of renal diseases consisting of CKD and AKI. However, extensive studies are still required to clarify the mechanisms and additional actions of prostacyclin in various kidney disease models.

## TXA2 IN KIDNEY DISEASES

TXA2 is a powerful platelet activator, and is also considered as potent smooth muscle constrictor. In addition, it also serves as a vascular smooth muscle cell mitogen. TXA2 is generated by a sequential catalyzation of phospholipase A2, COXs and TXA2 synthase (TXAS). Under physiological conditions, TXA2 derived from platelets mainly depends on the activity of COX-1. COX-1 inhibitor could inhibit the production of TXA2 and attenuate the early decrease in GFR in endotoxemia-induced acute kidney injury [134]. However, the responsible isoform of COXs for the formation of TXA2 during pathological conditions is still unknown [9]. TXAS is an endoplasmic reticulum membrane protein which is presented in different cells including smooth muscle cells, macrophages, platelets, endothelial cells, and kidney cells. TXAS expression can be regulated by a variety of factors. ONO-1301, a novel sustained-release prostacyclin analogue, was identified to inhibit TXAS expression [129].

Two subtypes of TP receptors, TPα and TPβ, have been proved to exist in various localizations of kidney and many other organs along with TXAS. In the kidney, TP receptors express in mesangial cells, podocytes, arterial vessel walls, luminal membranes of thick ascending limb of the Henle’s loop, and basolateral membranes of distal convoluted tubules and connecting tubules [135]. Generally speaking, TXA2 commonly couples with TP receptors and communicates mainly with Gq and G12/13, resulting in phospholipase C activation and Rho-GEF activation, respectively. To better understand the TXA2 on cellular signaling transduction, there is a nice review in the literature [136]. Although TPα and TPβ differ only in their C-terminal regions, several literatures had shown the differences of functions between TPα and TPβ, suggesting that the downstream signaling pathways may differ between both TP isoforms [137, 138]. TPα acts as the major TP receptor regulated by prostacyclin and NO. Moreover, RhoA signaling mediated by TPα could be directly blunted by prostacyclin and NO through protein kinase (PKA/PKG-dependent phosphorylation), while signaling of TPβ was not directly affected by prostacyclin or NO [139].

TXA2, which has been well established as efficacious activator of RhoA, was proved in detail to induce cell proliferation, differentiation, and inflammation, possibly through the mechanisms associated with AP-1, NF-KB, MRTF-A, and YAP [140]. TXA2 also played a critical role in the regulation of renal hemodynamics, which was determined by use of the TP agonist (U-46,619) [141, 142]. Patients with atrial fibrillation (AF) had a less decline in eGFR with the use of aspirin which inhibited TXA2 production [143]. One previous study reported that increased level of vascular ceramide induced by AngII contributed to the pathogenesis of renal injury possibly through TXA2-mediated vasoconstriction [144, 145]. Another study was also in line with this conclusion, showing that AngII/AT1 receptor/nSMase/ceramide-PLA2/TXA2 pathway contributed to the regulation of renal vasoconstriction [146]. In a model of type 2 diabetes, a TXA2 synthase inhibitor was beneficial in reducing glomerular lesion and proteinuria [147]. In addition to these findings, former study showed that inhibition of TXA2 synthase, pharmacological blockade of TP receptors, or genetic disruption of TP receptor could ameliorate the LPS-induced GFR decrease [148]. The reduction in GFR in response to endotoxemia was found to be resulted from the increased generation of TXA2 via COX-1 [134]. Additionally, evidence was also provided that COX-2 expressed in the macula densa regulated tubuloglomerular feedback through the generation of TXA2 and nNOS-dependent NO [149].

Beyond the role in renal vasculature, inhibition of TP receptors could rescue the kidney impairment to some degree [141]. Functions of TXA2 in mesangial cells in the regulation of growth response, matrix synthesis, glomerular thrombosis and fibrosis have been studied [147, 150]. Furthermore, in the kidney of diabetic apolipoprotein E KO mice, an antagonist of TP receptors (S18886) attenuated albuminuria, suppressed oxidative stress and inflammation, and blocked extracellular matrix deposition by activating MnSOD [151]. In accordance with this, in a CKD model of renal mass reduction, reduced renal mass led to microvascular remodeling and enhanced ET-1-induced mitochondrial ROS production and vascular contraction. All these abnormalities were related to the activation TP receptors [152]. With the treatment of puromycin amino nucleoside (PAN) or adriamycin, TXA2 was also increased in accordance with the podocyte damage. It was shown in this study that the podocyte injury was ameliorated by the TP receptor antagonist (SQ29548) or TP gene deletion [101]. In addition to this, some other studies also demonstrated the pathogenic roles of TXA2 and TP receptors in lupus nephritis [153], cyclosporine nephrotoxicity [154], and renal allograft rejection [155]. Overall, activation of TXA2/TP receptors can lead to the renal vasoconstriction, oxidative stress, and inflammation, which were involved in the onset and progression of kidney diseases.

## PGD2 IN KIDNEY DISEASES

Prostaglandin D2 is known for its involvement in a variety of neurophysiological functions including the body temperature control, hormone release and the sleep-wake cycle. In the kidney, with intrarenal infusion of PGD2, renal artery flow, urine output, creatinine clearance, and sodium and potassium excretion all increased dose-dependently [156]. Similar to other types of PGs, PGD2 is converted by prostaglandin D synthase (PGDS) through the common route of the PGs synthesis. Lipocalin type prostaglandin synthase (L-PGDS), also named beta trace protein (BTP), and Hematopoietic prostaglandin D synthase (H-PGDS) are two types of PGDS. They are different from each other in terms of cellular or tissue distribution and functional relevance [157]. H-PGDS is localized mostly in inflammatory cells, especially antigen-presenting cells and mast cells. H-PGDS has both pro-inflammatory and anti-inflammatory actions under different conditions [158, 159]. Fundamentally different from H-PGDS, L-PGDS can be detected in lots of tissues including the brain, heart, kidney, and lung [160]. During the recent years, as the biomarker in large population-based study cohorts, L-PGDS has aroused wide concern [161, 162]. To date, numerous investigations have shown that L-PGDS has emerged as a novel intracellular marker of GFR better than serum creatinine level [163–167]. Besides indicating the glomerular filtration rate, L-PGDS has also been identified as a proximal tubule damage marker during kidney injures [168]. Since its sensitivity in measuring GFR reduction, L-PGDS is becoming an important indicator of the outcome in kidney diseases. It captures the risks associated with decreased kidney function or pathophysiologic processes. Thus, it may provide an opportunity for the early diagnosis and early therapy in patients with kidney diseases [169].

L-PGDS is commonly regarded as a multiple function protein, acting as the PGD2 synthase and functioning as a lipophilic ligand-binding protein after its secretion. The protective role of L-PGDS has been studied in various experiment models, especially in cardiovascular and renal systems [170–172]. Details of L-PGDS in the cardiovascular system are beyond the scope of this review. In this review, we focused on the functions of L-PGDS in the kidney. It was clearly demonstrated that L-PGDS expression in the tubular epithelium remarkably increased in obstructed kidneys. The tubulointerstitial fibrosis caused by UUO was significantly suppressed in L-PGDS-KO mice, which stressed a critical role of L-PGDS in renal fibrosis [170]. However, other investigators showed that L-PGDS KO mice developed glomerular hypertrophy, fibrosis, basement membrane thickening, and high TGF-β deposition [172]. Function of L-PGDS in early stage diabetic nephropathy in rats and adriamycin-induced nephropathy in mice further suggested a possible contribution of PGD2 in chronic kidney diseases [173, 174]. The diversity of renal consequences suggested complex roles of L-PGDS in the kidneys challenged with different insults.

PGD2 interacts with two receptors, the DP1 receptor and DP2 receptor (also termed as CRTH2). PGD2 activates the DPs which then increase the level of cAMP. DP1 receptor appears to have various functions and is more widely expressed. In contrast, DP2 receptor mostly located in the inflammatory cell types such as Th2 cells, acting as a chemo-attractant receptor-homologous molecule. Roles of DP1 have been studied in cutaneous and pulmonary venous vasodilatation [175, 176], platelet aggregation [177], and mucin secretion [178]. DP2 receptor is not structurally the same as the DP1 and belongs to the family of chemokine receptors, contributing to the production of Th2 cytokines, such as IL-4, IL-5 and IL-13 [170, 179]. PGD2 receptors have not been studied that much in the kidney as in respiratory system [175, 180, 181]. However, studies have indicated that CRTH2 may involve in the progression of tubulointerstitial fibrosis and inflammation [182]. Researchers blocked CRTH2 by generating CRTH2-KO mice or using CRTH2 antagonist (CAY10471) in UUO model, and found that interstitial collagen deposition, collagen I gene expression and soluble collagen content were significantly suppressed in CRTH2-KO UUO mice. Furthermore, they revealed that PGD2 mediated the activation of Th2 lymphocytes through combining with CRTH2, which promoted the fibrosis via the production of IL-4 and IL-13 [170]. A previous study also suggested a DP receptor-independent function of PGD2 in kidney diseases [183]. PGD2 can be metabolized to biologically active J-series cyclopentone PGs, especially the 15d-PGJ2 which is considered as a natural endogenous ligand of PPARγ. Activation of PPARγ could effectively inhibit TGF-β-induced profibrotic effects in many organs. Thus, PGD2 may inhibit fibrosis by its end product of 15d-PGJ2 to activate PPARγ, restrain AP-1 and NF-κβ transcription factors. In addition, 15d-PGJ2 could also inhibit the expressions of CXCL9, CXCL10, and CXCL11 in HK-2 cells treated with combined IFN-γ and TNF-α [184]. Together, although the roles of PGD2 in renal system has not been fully understood, above findings still implied the importance of PGD2-generating cascade in the pathophysiology of kidney diseases.

## PGF2α IN KIDNEY DISEASES

PGF2α is also one of the major colooxygenase-mediated arachidonate metabolite in the kidney. PGF2α can be derived from either PGH2 via the PGF synthase or enzymatic conversion of PGE2 to PGF2α by PGE9 ketoreductase [185]. The cellular effects of PGF2α are mediated by G protein-coupled transmembrane receptors, namely FP_A_ and FP_B_. FP receptors are found to be linked to the increase of intracellular Ca2+ in response to PGF2α in renal cells, participating in the transformation of kidney fibroblasts [186]. Commonly, the FP receptors can be detected in the kidney. FPs were found to have high expression in the principal cells of the collecting duct, distal convoluted tubule, and podocytes of the glomeruli with less level in the thick ascending limb [187, 188]. Unlike other PGs, little is known about PGF2α in the renal diseases. Previous studies have shown that PGF2α is associated with natriuresis and diuresis through the activation of FP receptors in the cortical collecting duct [189, 190]. PGF2α inhibited the basolateral 40 pS K channels at high concentrations and stimulated these channels at low concentrations because of the different activation of FP_A_ and FP_B_ in PKC and Rho pathway, respectively [191]. FP activation could also inhibit arginine vasopressin-stimulated water permeability without increasing intracellular Ca^2+^ in cortical collecting ducts [189]. In animals, it was shown that FP KO mice exhibited modest polyuria and polydipsia with a mild defect in regulating renal medullary osmolality during water deprivation. This research also indicated that FP deletion reduced blood pressure through the inactivation of RAS, despite a compensatory enhancement of AT1 receptors and an augmented hypertensive response to AngII infusion [190]. In a renovascular hypertensive rat (RHR) model, ROS was considered as an initiator that activates endothelial COX-2 to form PGF2α to participate in endothelial dysfunction in the rabbit arteries. Treatment with celecoxib or tempol reduced blood pressure, increased renal blood flow, and restored endothelial function in renovascular hypertension rats, providing a new insight into understanding renal-related hypertension [192]. With respect to the role of PGF2α in oxidative stress, the production level of PGF2α has been shown to be changed dramatically during inflammatory response [193]. In a study carried out in autosomal dominant polycystic kidney disease (ADPKD) patients, increased serum concentrations of the oxidative stress markers of 8-isoprostane and PGF2α were associated with the GFR and kidney volume, suggesting a relationship between PGF2α and renal function during the growth of cystic kidney. Meanwhile, PGF2α was also confirmed to increase serum levels of MMP-1 and MMP-9 in this study [194]. Due to the abundance of PGF2α and its receptors in the kidney, there must be more functions in the renal incidences that have not been figured out. Thus, further investigation has to be conducted to fill the vacancy.

## CONCLUSIONS

In summary, PGs exert various functions in the pathology and physiology of kidney. The levels of PGs can be regulated at multiple steps. Among these steps, COXs and prostanoid synthases are of importance in the control of PGs’ production. PGs act by combining to their specific receptors or crosstalk with other receptors. Studies have shown that PGs exert multiple physiological functions in the kidney, such as maintaining glomerular filtration, modulating water and sodium excretion. Moreover, PGs are also involved in the pathology of various renal diseases including CKD and AKI. Strategies targeting any step of the COXs/PG synthases/PGs/receptors cascade might be potentially effective for the treatment of kidney diseases. Although there are still many problems and controversies in understanding the PGs system in kidney pathology, a number of current studies already provided valuable insights into the in-depth investigation of this field in the future.
